# Optic Nerve Head Morphological Changes Over 12 Hours in Seated and Head-Down Tilt Postures

**DOI:** 10.1167/iovs.61.13.21

**Published:** 2020-11-13

**Authors:** Laura P. Pardon, Han Cheng, Pratik Chettry, Nimesh B. Patel

**Affiliations:** University of Houston, College of Optometry, Houston, Texas, United States

**Keywords:** optic nerve head, minimum rim width, intraocular pressure, optic nerve sheath diameter

## Abstract

**Purpose:**

The purpose of this study was to determine changes in optic nerve head (ONH) morphology in seated and 6° head-down tilt (HDT) postures over a 12-hour period.

**Methods:**

Thirty eyes of 30 healthy human subjects (15 females) were included. Composite radial and circular optical coherence tomography (OCT) scans centered on the ONH, intraocular pressure (IOP), and optic nerve sheath diameter (ONSD) were acquired every two hours from 7 a.m. to 7 p.m. for both seated (*n* = 30) and HDT (*n* = 10) sessions. Global minimum rim width (BMO-MRW), total retinal thickness (TRT), retinal nerve fiber layer thickness (RNFLT), and Bruch's membrane opening (BMO) height were quantified.

**Results:**

BMO-MRW decreased an average of 9.55 ± 8.03 µm (*P* < 0.01) over 12 hours in a seated position (range, −26.64 to +3.36 µm), and thinning was greater in females (−13.56 vs. −5.55 µm, *P* = 0.004). Modest decreases in TRT from the BMO to 500 µm (*P* < 0.04) and RNFLT for the 2.7, 3.5, and 4.2 mm circular scans (*P* < 0.02) were also observed. BMO-MRW thinning was not related to changes in IOP or ONSD (*P* = 0.34). In HDT, IOP and ONSD increased, BMO height moved anteriorly, and BMO-MRW thinning did not occur (*P* > 0.1).

**Conclusions:**

The neuroretinal rim thins throughout the day in healthy individuals, and this change cannot be explained by changes in IOP or ONSD during the same time period. A HDT posture blunts the neuroretinal rim thinning observed in a seated position, suggesting a role of the translaminar pressure difference.

The optic nerve head (ONH) is a relative weak point in the scleral shell and, as a result, is susceptible to the effects of pressures acting on the globe. Intraocular pressure (IOP) and retrobulbar cerebrospinal fluid pressure (CSFP) represent two opposing forces on the ONH, and the translaminar pressure difference (IOP minus CSFP) is thought to have important implications for optic neuropathies such as glaucoma and papilledema.^1^ Quantification of ONH morphology, using optical coherence tomography (OCT), is sensitive for detecting these conditions,^2^^−^^4^ and OCT thickness measures have generally exhibited excellent repeatability.^5^^−^^9^ Clinically, OCT metrics are considered to be static; however, there is significant evidence from human and animal studies demonstrating that ONH structure is sensitive to transient changes in IOP and CSFP; a relative increase in IOP results in neuroretinal rim thinning and posterior ONH deformation, whereas a relative increase in CSFP results in neuroretinal rim thickening and anterior ONH deformation.^10^^−^^13^

Intraocular pressure and CSFP are not fixed, but exhibit fluctuations throughout the day. Normal diurnal variation in IOP is approximately 3-4 mmHg in a seated position, with highest pressures occurring in the early morning.^14^ Less is known regarding the diurnal variation in CSFP due to the invasive nature of measurement, however, intracranial pressure has been found to vary approximately 5 mmHg in non-human primates using wireless telemetry.^15^ In addition to normal diurnal fluctuations, IOP and CSFP are known to change with body posture; transitioning from a seated to a supine or head-down tilt (HDT) posture results in a greater increase in CSFP than IOP.^16^^,^^17^ For example, CSFP is typically slightly negative to 0 mmHg in a seated position and increases to about 10–12 mmHg in a supine position, whereas IOP only increases from about 15 to 17 mmHg with the same change in posture.^16^^,^^18^ While fluctuations in pressures are habitually experienced in our daily lives, whether these normal changes in IOP and CSFP are reflected in ONH morphology, and the normal diurnal variability of ONH measures, remains unknown.

The goal of the present study was to determine how ONH morphology is influenced by time of day and body posture in young, healthy individuals. We hypothesized that ONH morphology would reflect changes in the translaminar pressure difference and investigated normal changes in ONH morphology over a 12-hour period and, for a subset of subjects, how ONH morphology is altered over time in a 6° HDT posture (i.e., a condition of relatively elevated retrobulbar CSFP). In order to relate structural changes to changes in pressures acting on the ONH, IOP and optic nerve sheath diameter (ONSD), a surrogate measure that responds rapidly to changes in CSFP in vivo,^19^ were quantified at each imaging session.

## Methods

Thirty healthy human subjects between ages 18 to 40 years old, with no history of ocular pathology, were recruited at the University of Houston College of Optometry. Written informed consent was obtained from all subjects after explaining the nature and possible consequences of the study. The study protocol was reviewed by the Committee for Protection of Human Subjects at the University of Houston and adhered to the tenets of the Declaration of Helsinki.

All 30 subjects participated in the 12-hour seated portion of the experiment to determine normal changes in ONH morphology throughout the day. Subjects were asked to report to the lab at 7 a.m., at which time a brief ocular/systemic health questionnaire, visual acuity, standard automated perimetry, and fundus photography were obtained to confirm good ocular health of all subjects. Data collection occurred over a 12-hour period, with seven sessions in total (7 a.m., 9 a.m., 11 a.m., 1 p.m., 3 p.m., 5 p.m., 7 p.m.). At each session, OCT, rebound tonometry, B-scan ultrasonography, blood pressure (BP), and optical biometry were obtained with subjects in a seated position.

Retrobulbar CSFP and IOP are at their lowest values in a seated or upright posture and increase with increasing degrees of body tilt.^16^^,^^20^ Because CSFP increases to a greater extent than IOP with increasing amounts of tilt, the translaminar pressure difference is at a maximum in a seated/upright posture and decreases as tilt increases.^16^^,^^17^ A 6° HDT posture was selected for the present study to investigate the effects of an altered translaminar pressure difference, favoring elevated CSFP, on ONH structure over time.

A subset of 10 subjects participated in the 12-hour 6° HDT experiment on a separate day. Similar to the seated experiment, subjects presented to the lab at 7 a.m. First, baseline OCT, rebound tonometry, B-scan ultrasonography, BP, and optical biometry measures were obtained with subjects in a seated posture. Subjects then assumed a 6° HDT posture by lying on a surgical bed adjusted to a −6° angle so that the subject's feet were elevated relative to the head. In this position, OCT, rebound tonometry, B-scan ultrasonography, and BP were repeated to obtain approximately 7 a.m. HDT measures. As for the seated experiment, OCT, rebound tonometry, and ultrasonography were repeated every two hours through 7 p.m.; BP was measured every 30 minutes to ensure subject safety in the HDT position. Subjects remained in HDT from 7 a.m. through 7 p.m. with allowed breaks totaling up to 36 minutes, ensuring that subjects were in an HDT position for at least 95% of the 12-hour period. After completing the 7 p.m. session, an endpoint seated scan session was performed, repeating the same testing obtained for the 7 a.m. seated session.

### Optical Coherence Tomography Scan Acquisition

**Figure 1.**
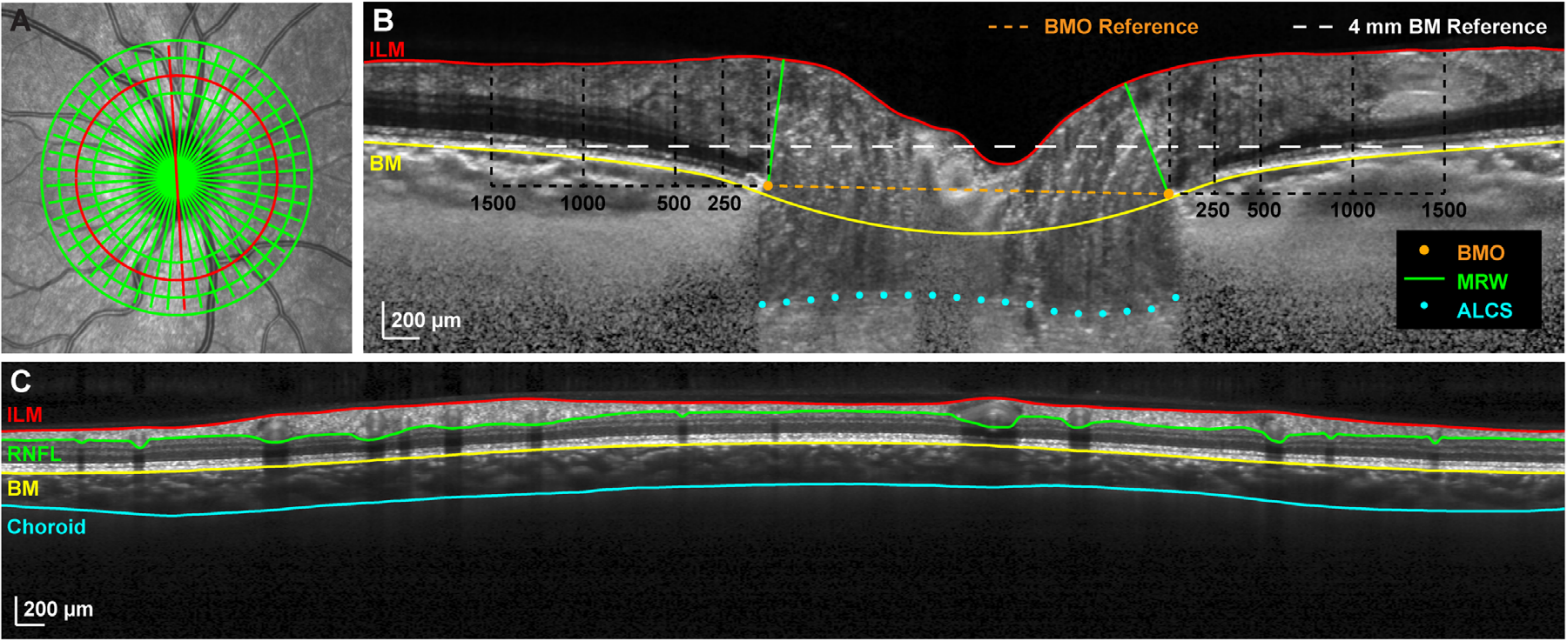
Optical coherence tomography segmentation and parameters. (**A**) A 20° SLO image illustrating the custom composite OCT scan consisting of a 24-line radial scan and four circular scans (diameters 2.7, 3.5, 4.2, and 4.9 mm) centered on the ONH. (**B**) Compensated radial B-scan corresponding with the location of the *red line* in **A**. ILM and BM segmentations, manually selected BMO and ALCS points, BM and BMO reference planes, and locations of concentric annuli for TRT quantification (BMO to 250 µm, 250 to 500 µm, 500 to 1000 µm, and 1000 to 1500 µm) are shown. (**C**) Circular B-scan corresponding with the location of the 3.5 mm diameter *red circle* in **A** and illustrating ILM, RNFL, BM, and choroid segmentations.

Optical biometry (Lenstar LS900; Haag-Streit, Koeniz, Switzerland) was acquired prior to the baseline OCT scan, and corneal curvature values were entered into the OCT system for accurate transverse scaling. At each session, a custom composite OCT scan consisting of a 24-line (15°) radial scan and four circular scans (diameters of 2.7, 3.5, 4.2, and 4.9 mm) centered on the ONH was obtained using the Glaucoma Module Premium Edition software and Spectralis OCT2 system (Heidelberg Engineering, Heidelberg, Germany; Fig. 1A) without pupillary dilation; a second scan was obtained at the 7 a.m. session to assess repeatability of OCT measures. The Spectralis Flex Module, consisting of an OCT system attached to a surgical arm, was used for scans while subjects were in a HDT position. Additionally, the anatomic positioning system (APS) and AutoRescan feature were used for both seated and HDT experiments to ensure that follow-up scans were accurately scaled and aligned. Scans were repeated if they were not adequately centered (e.g., if the image was cut off or if substantial tilt was present) or if they were of poor quality (less than approximately 30 dB). One eye was selected at random for data analysis, and the same eye was analyzed for both seated and HDT experiments. Raw scan data (*.vol files) were exported and analyzed using programs written in Matlab (The Mathworks, Natick, MA, USA).

### Optic Nerve Head Analysis

For each radial B-scan, the internal limiting membrane (ILM) was corrected for segmentation errors, and Bruch's membrane (BM) was delineated. After image compensation,^21^ the points corresponding with BM opening (BMO) and the anterior lamina cribrosa surface (ALCS) were manually selected (Fig. 1B). The ALCS was only traced in regions where the anterior boundary was clearly defined, and this boundary was detectable in the majority of radial B-scans for each subject.

The neuroretinal rim minimum rim width (BMO-MRW) was quantified as the minimum distance from the selected BMO points to the ILM, constrained to within the BMO region. A best-fit ellipse to the BMO points was used to quantify the BMO area. The relative anterior-posterior position of the BMO was quantified as the parameter BMO height, the minimum distance from a 4 mm BM reference line centered on the ONH to the BMO.^4^^,^^22^ BMO-ALCS depth (BMO-ALCSD) was quantified as the average perpendicular distance between the marked lamina and a line connecting the BMO points, using only data within the central 50% of the region between the BMO points to minimize the effects of peripheral curvatures in the laminar surface. To assess the circumpapillary tissue, a total retinal thickness map for the region scanned was generated using linear interpolation, and subsequently average thickness values were quantified for annular bands at eccentricities corresponding to the following: (1) BMO to 250 µm (TRT250), (2) 250 to 500 µm (TRT500), (3) 500 to 1000 µm (TRT1000), and (4) 1000 to 1500 µm (TRT1500).^4^^,^^23^

### Circumpapillary Retinal Nerve Fiber Layer and Choroid Analysis

For each circular B-scan, ILM, RNFL, and BM segmentations were manually corrected for segmentation errors, and the choroid/sclera interface was manually delineated following image compensation (Fig. 1C).^21^ From these scans, average circumpapillary RNFL thickness and choroid thickness were determined. Additionally, total retinal thickness was quantified to facilitate comparisons with RNFL thickness.

### Ultrasonography and Optic Nerve Sheath Diameter Measurement

Direct assessment of CSFP was not performed in this study, as such measures are invasive in nature and not indicated in healthy individuals. The retrobulbar ONSD changes rapidly in response to alterations in CSFP and was used as a surrogate for relative changes in CSFP over the 12-hour study period.^19^ Ultrasonography images were acquired from the contralateral eye using a 10 MHz B-scan probe (Aviso S; Quantel Medical, Clermont-Ferrand, France) at each session. Although care was taken to apply minimal pressure to the globe when obtaining images, the contralateral eye was selected to avoid any confounding effects that mild pressure application to the globe may have on ONH measures. In theory, any change in CSFP should be reflected in the ONSD of both eyes.

**Figure 2.**
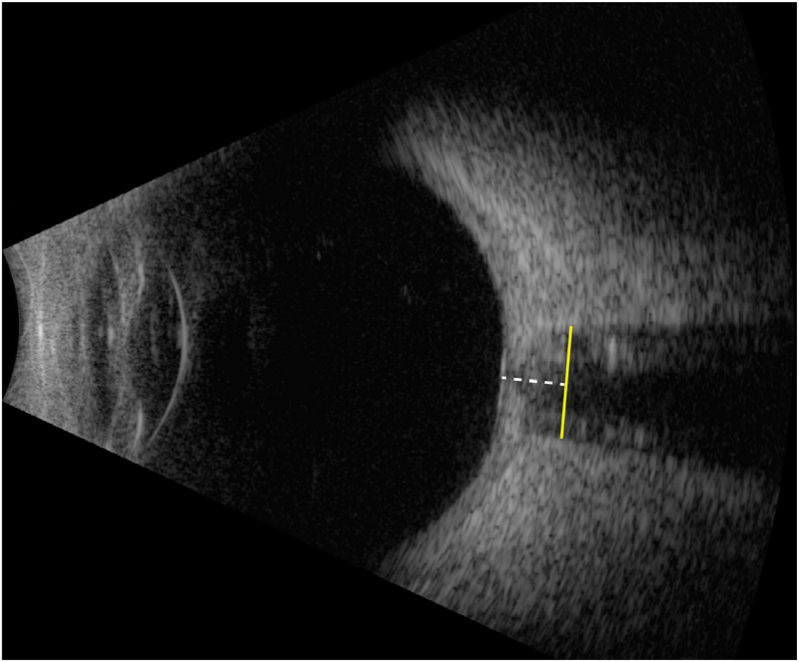
Ultrasonography measurement of optic nerve sheath diameter. Vertical axial B-scan demonstrating good centration of the optic nerve and distinct optic nerve sheath boundaries. ONSD (*yellow line*) was quantified 3 mm (*white dashed line*) posterior to the globe.

Vertical axial B-scans were performed over the eyelid with the subject looking straight ahead. For each session, several scans were obtained, and the frame with the best quality was used for ONSD quantification. Quality B-scans demonstrated a well-centered optic nerve extending from the posterior globe and parallel and distinct optic nerve sheath boundaries and represented the widest diameter in the absence of artifact. ONSD was measured 3 mm behind the globe using the software's calipers (Fig. 2).^24^^,^^25^

### Intraocular Pressure and Ocular Perfusion Pressure

At each session, IOP was quantified as the average of three measures obtained using the ICare rebound tonometer (ICare, Helsinki, Finland). When obtaining IOP measures in a HDT position, subjects rotated their entire body to the left without elevating their head so that they were in a left lateral decubitus position; this method was selected to avoid possible compression of the jugular veins, which may occur when the head alone is rotated and could impact IOP measures.

Blood pressure was assessed using an automated cuff (Omron HEM-907XL; Omron Healthcare, Inc., Lake Forest, IL, USA) over the brachial artery, and the average of two measures, taken a minute apart, was used for analysis. Average blood pressure and IOP for each session were used to calculate mean arterial pressure (MAP) and mean ocular perfusion pressure (MOPP). Direct measures of MOPP would require invasive techniques; instead, MOPP was estimated by taking into account the vertical distance between the eye and the cuff (i.e., the change in hydrostatic pressure^26^^,^^27^) in a seated vs. HDT posture using the following equations:

(1) MAP = (2 × diastolic BP + systolic BP)/3
(2) MOPP = [MAP – (0.77 mmHg/cm × vertical distance from cuff to eye)] – IOP.

### Statistical Analysis

Global values for all parameters were used for statistical analysis and are expressed as mean ± standard deviation. For each parameter, equal variance was confirmed using a Bartlett's test in Minitab 18 (Minitab, Inc., State College, PA, USA). Using GraphPad Prism 7 (GraphPad Software, Inc., San Diego, CA, USA), one-way ANOVA with repeated measures was performed to determine whether each parameter significantly changed over the 12-hour period; a Dunnett's multiple comparison's test was then used to compare each time point to the 7 a.m. seated baseline for the same day. Paired *t*-tests were used to compare baseline values on the days of the seated versus HDT experiments and changes in RNFL versus total retinal thickness measures obtained from the same circular scans, and unpaired *t*-tests compared differences between sexes. Linear regression analysis was performed to determine relationships between parameters, and a Sidak's multiple comparisons test was used to compare change in parameters from baseline in a seated versus HDT posture for the 10 subjects who participated in both experiments (9 a.m. through 7 p.m.). Additionally, multiple regression analysis was performed using SigmaPlot 12.0 (Systat Software, Inc., San Jose, CA, USA) to investigate whether changes in BMO-MRW are related to changes in IOP and ONSD. Statistical significance was set at alpha = 0.05 for all analyses.

To assess repeatability of OCT parameters, within-subject standard deviation (Sw) and coefficient of variation (CV) were determined using 7 a.m. seated data for all subjects who had two OCT scans acquired at this time point on the day of the 12-hour seated study (*n* = 24). Repeatability was calculated as 2.77*Sw (Table 2).^28^

## Results

**Figure 3.**
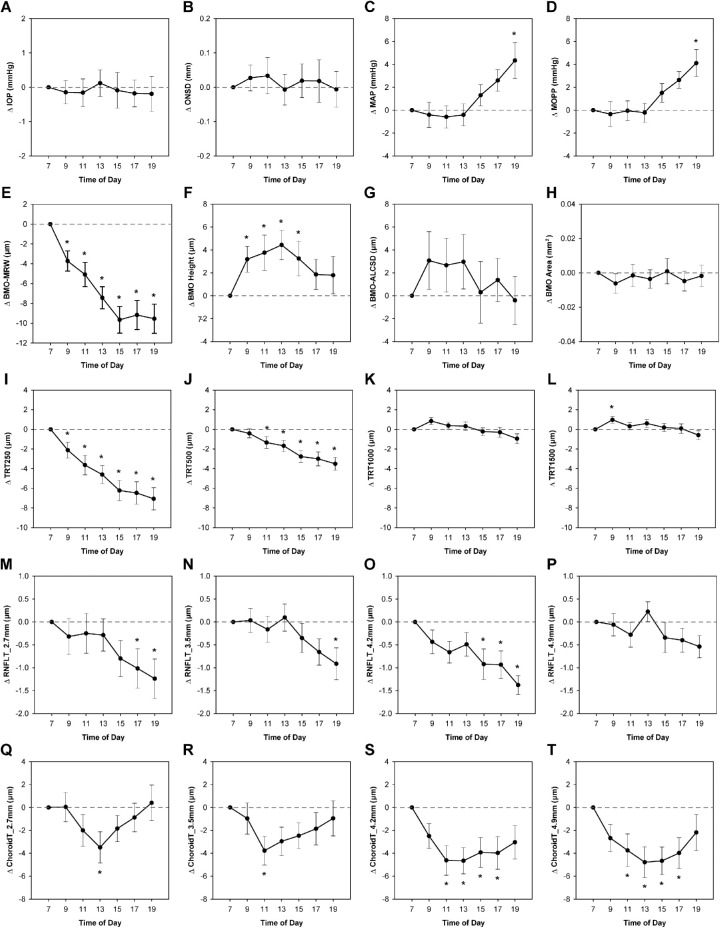
Changes in parameters over a 12-hour period in a seated position. Plots depict mean change ± standard error from the 7 a.m. baseline for parameters obtained during the seated experiment (*n* = 30). Parameter values at each time point were compared to those obtained at the 7 a.m. baseline, and statistically significant differences are denoted with an *asterisk* (*P* < 0.05). RNFLT, retinal nerve fiber layer thickness; ChoroidT, choroid thickness.

**Figure 4.**
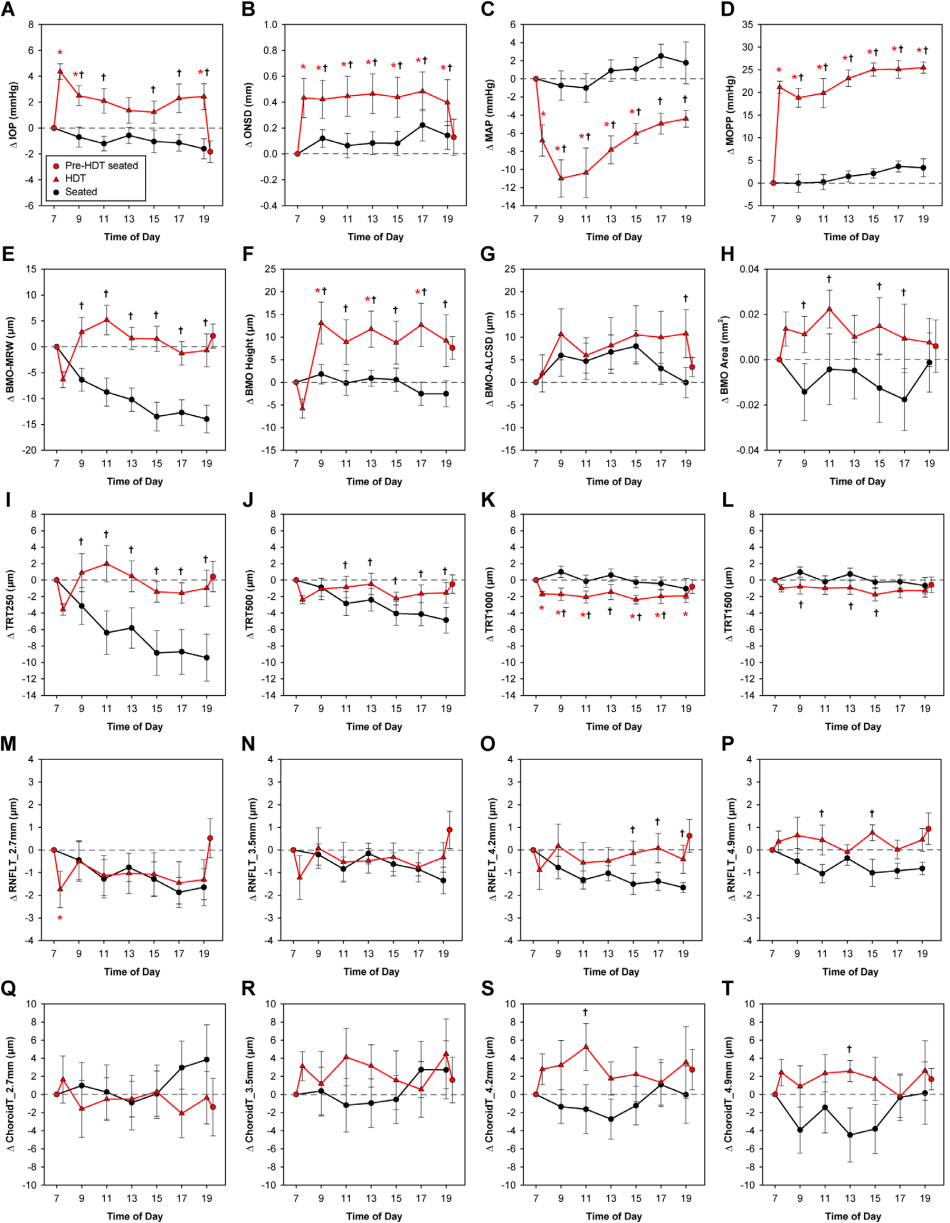
Changes in parameters over 12 hours in a head-down tilt versus seated position. *Red plots* depict mean change ± standard error from the 7 a.m. seated baseline for parameters obtained during the HDT experiment (*n* = 10). *Circles* represent data obtained in a seated position, and *triangles* represent data obtained in an HDT position. Parameter values at each time point were compared to those obtained at the 7 a.m. seated baseline, and statistically significant differences are denoted with a *red asterisk* (*P* < 0.05). Additionally, *black plots* show data obtained over 12 hours in a seated position for the same 10 subjects; a significant difference in change from baseline between the seated and HDT conditions is denoted with a *black dagger*. RNFLT = retinal nerve fiber layer thickness; ChoroidT = choroid thickness.

All 30 subjects participated in the 12-hour seated experiment, and a subset of 10 subjects also participated in the 12-hour HDT experiment. Subject demographics are shown in Table 1^2^. Figures 3 and 4 depict changes in each parameter from the 7am seated baseline for the seated (n = 30) and HDT vs. seated (n = 10) conditions, respectively. Mean baseline values for each experiment are shown in Table 3. The following sections first describe changes in pressures to which the ONH is exposed, followed by changes in ONH structure over 12-hour periods in seated and HDT postures.

**Table 1.** Subject Demographics

	Seated	HDT and
	Experiment	Seated Experiments
	(*n* = 30)	(*n* = 10)
Sex		
Number of males	15	4
Number of females	15	6
Age (years)	29.3 ± 4.1	29.5 ± 4.8

**Table 2.** Within-Subject Repeatability of OCT Parameters Obtained at 7 a.m. on the Same Day

	Repeatability^*^	Coefficient of
	(*n* = 24)	Variation (%)
BMO-MRW (µm)	4.40	0.45
BMO Height (µm)	8.03	2.88
ALCSD (µm)	25.01	2.08
BMO Area (mm^2^)	0.06	0.80
TRT250 (µm)	4.85	0.45
TRT500 (µm)	3.07	0.30
TRT1000 (µm)	2.13	0.23
TRT1500 (µm)	2.80	0.32
RNFLT_2.7 (µm)	3.82	0.97
RNFLT_3.5 (µm)	2.60	0.79
RNFLT_4.2 (µm)	2.41	0.85
RNFLT_4.9 (µm)	2.24	0.90
ChoroidT_2.7 (µm)	20.28	4.01
ChoroidT_3.5 (µm)	13.85	2.35
ChoroidT_4.2 (µm)	13.71	2.17
ChoroidT_4.9 (µm)	14.76	2.22

ChoroidT = choroid thickness.

* Calculated as 2.77*Sw.

**Table 3.** Baseline 7 a.m. Seated Values For Seated and HDT Experiments

	Seated (*n* = 30)^*^	Seated (*n* = 10)	HDT (*n* = 10)
IOP (mmHg)	13.50 ± 3.14	14.87 ± 3.48	15.07 ± 2.49
ONSD (mm)	5.07 ± 0.49	4.78 ± 0.48	4.82 ± 0.47
MAP (mmHg)	87.51 ± 9.46	85.60 ± 9.70	88.10 ± 9.02
MOPP (mmHg)	44.45 ± 10.36	41.47 ± 10.25	43.77 ± 10.52^†^
BMO-MRW (µm)	355.31 ± 61.76	368.32 ± 87.52	371.38 ± 90.21
BMO Height (µm)	−102.01 ± 45.59	−103.70 ± 38.25	−106.79 ± 41.49
ALCSD (µm)	−440.14 ± 90.69	−436.94 ± 87.78	−432.50 ± 91.62
BMO Area (mm^2^)	2.04 ± 0.44	1.91 ± 0.54	1.88 ± 0.57
TRT250 (µm)	394.77 ± 36.66	404.46 ± 51.81	404.18 ± 47.96
TRT500 (µm)	375.02 ± 20.64	377.20 ± 26.52	377.76 ± 24.26
TRT1000 (µm)	343.08 ± 13.27	339.86 ± 12.53	340.69 ± 12.09
TRT1500 (µm)	317.12 ± 11.22	313.00 ± 10.59	312.54 ± 9.84
RNFLT_2.7 (µm)	142.85 ± 14.51	141.54 ± 19.71	142.81 ± 18.78
RNFLT_3.5 (µm)	118.96 ± 10.79	117.33 ± 12.82	117.68 ± 13.02
RNFLT_4.2 (µm)	102.93 ± 9.10	101.58 ± 10.51	100.68 ± 10.55
RNFLT_4.9 (µm)	90.10 ± 7.91	88.90 ± 8.87	87.65 ± 9.59
ChoroidT_2.7 (µm)	179.84 ± 41.48	181.72 ± 34.41	188.18 ± 33.44
ChoroidT_3.5 (µm)	211.25 ± 50.72	212.87 ± 39.84	213.59 ± 39.09
ChoroidT_4.2 (µm)	228.83 ± 53.85	233.54 ± 43.27	232.96 ± 41.43
ChoroidT_4.9 (µm)	241.26 ± 55.44	246.36 ± 47.93	246.45 ± 45.83

* For MAP and MOPP, *n* = 26.

† Significant difference between seated (*n* = 10) and HDT (*n* = 10) baseline values (*P* < 0.05).

### Intraocular Pressure

For the seated Experiment, there was no significant change in IOP over the 12-hour period studied (*P* = 0.99, Fig. 3A). On the day of the HDT experiment, IOP immediately increased by 4.37 ± 1.92 mmHg when transitioning from a seated to HDT posture (*P* < 0.001). IOP remained elevated 1 to 4 mmHg above baseline over the 12 hours in HDT (Fig. 4A). After subjects returned to a seated position, IOP decreased to approximately baseline (*P* = 0.21). Change in IOP was significantly increased in HDT relative to a seated position at all time points except 1 p.m. (*P* < 0.04).

### Optic Nerve Sheath Diameter

Similar to IOP, there was no significant change in ONSD over the 12-hour period with subjects in in a seated position (*P* = 0.97, Fig. 3B). For the HDT experiment, ONSD increased by 0.43 ± 0.48 mm when transitioning from a seated to HDT posture (*P* < 0.001) and remained significantly elevated above the seated baseline value (*P* < 0.003, Fig. 4B). Change in ONSD was significantly increased in HDT relative to a seated position for all time points in HDT (*P* < 0.04). Following a return to a seated position, the ONSD rapidly returned to baseline (*P* = 0.75).

There is currently no accurate method for quantifying change in CSFP from change in ONSD; rather, ONSD is used in the present study to detect relative increases or decreases in CSFP over time. However, previous studies have investigated changes in CSFP with changes in body posture and can provide insight regarding likely changes in CSFP in our subjects. In a seated position, CSFP is approximately 0 mmHg or slightly negative.^16^^,^^18^^,^^20^ CSFP increases with reclining body posture, increasing to about 10 to 12 mmHg on average in a supine position and to an even greater extent in HDT.^16^^,^^18^ Therefore the change in retrobulbar CSFP in a HDT posture was likely considerably larger than the 1 to 4 mmHg increase in IOP observed in our subjects. Because ONSD remained increased over the entire duration in HDT, the translaminar pressure difference should be lower in HDT relative to a seated position over the 12-hour period.

### Blood Pressure and Ocular Perfusion Pressure

Blood pressure data were missing at one or more time points for four of the 30 subjects; therefore MAP and MOPP were calculated only for the 26 subjects with a complete data set. For the seated experiment, both MAP and MOPP were stable through 1 p.m., then gradually increased, and were significantly elevated relative to baseline at 7 p.m. (*P* < 0.005, Figs. 3C, 3D).

Four of the 10 subjects in the HDT experiment did not have a final BP reading in the seated position; therefore, BP data for the final 7 p.m. seated session was not included in the analysis for MAP or MOPP. In a HDT position, MAP initially decreased 6.80 ± 5.49 mmHg from its seated baseline value (*P* = 0.004), then gradually increased from 11 a.m. onward; MAP was significantly reduced relative to baseline from 7 a.m. through 3 p.m. (*P* < 0.02, Fig. 4C). When going from a seated to a HDT position, MOPP initially increased 21.15 ± 4.19 mmHg and remained elevated above baseline for all subsequent time points (*P* < 0.001, Fig. 4D). MAP was significantly reduced, and MOPP significantly increased, in HDT relative to a seated position for all time points (*P* < 0.02). Despite a lower MAP in a HDT position, this increase in MOPP is a result of the shortened (and negative) vertical distance between the BP cuff and the eye.

### Bruch's Membrane Opening and Anterior Lamina Cribrosa Surface Position

**Figure 5.**
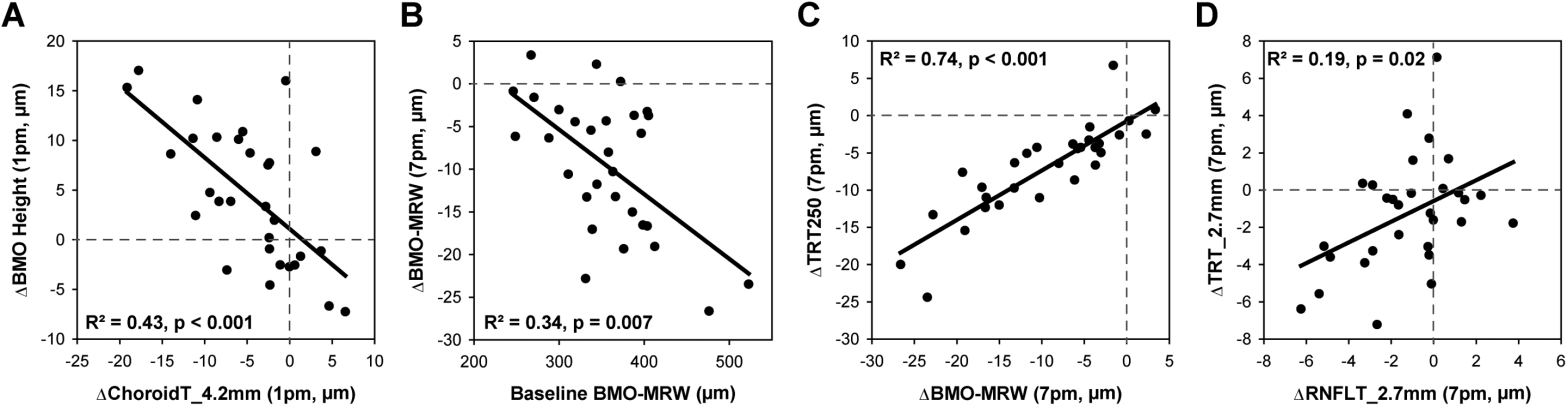
Relationships between OCT parameters. Linear regression relationships between (**A**) change choroid thickness (ChoroidT, 4.2 mm circular scan) and change in BMO height from 7 a.m. to 1 p.m., (**B**) baseline BMO-MRW and change in BMO-MRW from 7 a.m. to 7 p.m., (**C**) change in BMO-MRW and change in TRT250 from 7 a.m. to 7 p.m., and (**D**) change in RNFL thickness (RNFLT) and change in total retinal thickness (TRT) from 7 a.m. to 7 p.m. for the 2.7 mm circular scan.

For the seated experiment, there were no significant changes in ALCSD or BMO area over the 12 hours studied (*P* > 0.74, Figs. 3G, 3H). However, BMO height exhibited significant shortening of 3.7 µm on average from 9 a.m. through 3 p.m. (*P* < 0.05, Fig. 3F). This change in BMO height was similar in magnitude to a reduction in circumpapillary choroid thickness during the same period of time (Figs. 3Q–T). A decrease in choroid thickness would result in relative posterior displacement of the BM reference plane used to quantify BMO height. In fact, there was a significant relationship between change in choroid thickness for the 4.2 mm circle (the location closest to the 4 mm BM reference plane) and change in BMO height from 7 a.m. to 1 p.m., with *R*^2^ = 0.43 (*P* < 0.001, Fig. 5A). Thus the relative displacement of the BMO height is a reflection of underlying choroidal changes rather than a manifestation of increased CSFP.

There were no significant changes in BMO-ALCSD or BMO area over the 12-hour period in HDT (*P* > 0.15, Figs. 4G, 4H). After an initial posterior movement when assuming a HDT posture, BMO height demonstrated anterior displacement of about 9 to 13 µm that reached statistical significance at 9 a.m., 1 p.m., and 5 p.m. (*P* < 0.02, Fig. 4F). The BMO was more anteriorly displaced from baseline in a HDT position compared with a seated position at all time points (*P* < 0.02). In contrast to the seated arm of the study, changes in BMO height were not related to choroid thickness, suggesting that anterior displacement of the BMO may be a result of elevated CSFP.

### Minimum Rim Width

Based on the above findings that IOP and ONSD (and therefore presumably CSFP) do not significantly change throughout the day in a seated position, neuroretinal rim thickness would be also expected to remain stable. However, BMO-MRW thinned an average of 9.55 ± 8.03 µm by 7 p.m.; the majority of this change occurred between 7 a.m. and 3 p.m., after which no further change was observed (Fig. 3E). Females demonstrated greater BMO-MRW thinning than males on average (−13.56 ± 7.2 vs. −5.55 ± 6.9 µm, *P* = 0.004), despite similar baseline values (369.5 ± 16.8 vs. 341.1 ± 14.7 µm, *P* = 0.2) and changes in IOP and ONSD (*P* > 0.3). The subset of 10 subjects participating in the HDT experiment exhibited slightly greater BMO-MRW thinning, decreasing an average of 13.97 ± 8.40 µm; however, this was not significantly different than the decrease observed for all 30 subjects (*P* = 0.18). For both sets of seated subjects, BMO-MRW was significantly reduced from the 7 a.m. baseline at all subsequent time points (*P* < 0.005). Despite considerable inter-individual differences in the extent of BMO-MRW change over the 12-hour period, 27 of the 30 subjects in the seated experiment demonstrated BMO-MRW thinning at 7 p.m. relative to 7 a.m. (range, −26.64 to +3.36 µm). Subjects were also observed to have a wide range of baseline BMO-MRW values (246.01–522.61 µm). The BMO-MRW change from 7 a.m. to 7 p.m. was modestly related to baseline BMO-MRW (*R*^2^ = 0.34, *P* = 0.007, Fig. 5B). Because the translaminar pressure difference can influence ONH structure, multiple regression analysis for BMO-MRW, IOP, and ONSD was performed. Additionally, simple linear regression between BMO-MRW and MOPP was performed to determine whether changes in BMO-MRW were related to ONH perfusion. Relationships between changes in BMO-MRW, IOP, and ONSD, and between changes in BMO-MRW and MOPP, were not significant in a seated position (*P* > 0.07).

In a HDT posture, BMO-MRW did not significantly change from the 7 a.m. seated baseline over the 12-hour period, nor were there significant differences in BMO-MRW change between males and females at 7 p.m. (*P* > 0.11). The changes in BMO-MRW from baseline significantly differed between seated and HDT postures for all time points, with thinning observed in a seated position but not HDT (*P* < 0.001). Multiple regression analysis for the HDT experiment revealed a weak relationship between changes in BMO-MRW, IOP, and ONSD (*R*^2^ = 0.10, *P* < 0.01); although a change in BMO-MRW was inversely related to a change in IOP (*P* = 0.030), its relationship with change in ONSD did not reach statistical significance (*P* = 0.054). There was no relationship between change in BMO-MRW and change in MOPP (*P* = 0.53).

### Total Retinal Thickness

To determine whether the observed thinning at the neuroretinal rim extended to the adjacent peripapillary retina, total retinal thickness was determined at four annular eccentricities from the BMO. One-way ANOVA with repeated measures was performed separately for each annulus. Similar to BMO-MRW, TRT250 gradually decreased over time and was significantly reduced from baseline at all subsequent time points (p < 0.03), decreasing an average of 7.07 ± 6.21 µm by the end of the 12-hour period (Fig. 3I); there was a significant correlation between change in BMO-MRW and change in TRT250 from 7 a.m. to 7 p.m. (*R*^2^ = 0.74, *P* < 0.001, Fig. 5C). TRT500 was significantly reduced from 11 a.m. onward (*P* < 0.04), thinning an average of 3.50 ± 3.55 µm by 7 p.m. (Fig. 3J). However, there was no significant thinning of TRT1000 or TRT1500 from baseline over the 12-hour period (Figs. 3K, 3L). As with BMO-MRW, females exhibited greater thinning than males of TRT250 (−9.6 ± 1.6 vs. −4.6 ± 1.4 µm, *P* = 0.02) and TRT500 (−4.9 ± 0.9 vs. −2.1 ± 0.8 µm, *P* = 0.03).

For the HDT experiment, there was no significant change in TRT250, TRT500, or TRT1500 over the 12-hour period from the 7 a.m. seated baseline values (*P* > 0.17, Figs. 4I, 4J, 4L). TRT250 followed a similar pattern as BMO-MRW, and change in TRT250 significantly differed between seated and HDT postures for all time points, with thicker measures observed in HDT (*P* < 0.02). In contrast, TRT1000 and TRT1500 showed a trend toward thinning in HDT relative to a seated position.

Overall, the BMO-MRW and total retinal thickness findings show that while greater thinning occurs at the ONH, tissue thinning also extends into the peripapillary retina to an eccentricity of approximately 500 µm. Under normal conditions in an upright posture, these changes are not related to corresponding changes in IOP or ONSD at the same time points. However, under conditions of a decreased translaminar pressure difference, as occurs in HDT, the change in thickness measures is blunted. These data suggest that changes in ONH morphology occur over an extended time period, with decreases in BMO-MRW in a seated position likely reflecting the increase in the translaminar pressure difference experienced as one changes posture when transitioning from sleeping to waking states.

### Retinal Nerve Fiber Layer Thickness

Four circular scans of diameters 2.7, 3.5, 4.2, and 4.9 mm were used to quantify circumpapillary RNFL thickness. Based on a mean BMO area of 2.04 mm^2^ for subjects in this study, mean BMO diameter is approximately 1600 µm. As a result, the 2.7, 3.5, 4.2, and 4.9 mm scans would be located approximately 550, 950, 1300, and 1650 µm from the BMO on average. Therefore RNFL thickness was quantified at locations within the TRT1000 annulus, TRT1500 annulus, and just beyond the TRT1500 annulus.

RNFL thickness for the three innermost circles was significantly reduced by the end of the 12-hour period in a seated position (*P* < 0.02, Figs. 3M–O); however there was no significant difference between sexes (*P* > 0.06). To directly compare changes in RNFL with changes in total retinal thickness, total retinal thickness was quantified for each of the circular scans falling within the TRT1000 annulus (i.e., 2.7 and 3.5 mm). Similar to RNFL thickness, total retinal thickness for the innermost circle was significantly reduced at 7 p.m. (*P* = 0.03), and both RNFL and total retinal thickness decreased by similar amounts at this location (−1.24 ± 2.35 µm and −1.27 ± 3.04 µm, respectively; *P* = 0.95). Total retinal thickness for the 3.5 mm scan did not significantly decrease from baseline, however, change at 7 p.m. was similar between RNFL and total retinal thickness (−0.91 ± 1.91 and −0.69 ± 2.45, *P* = 0.67). These findings suggest that any changes in total retinal thickness may be due to changes occurring within the RNFL. There was a modest relationship between changes in RNFL and total retinal thickness from 7 a.m. to 7 p.m. for the 2.7 mm circle (*R*^2^ = 0.19, *P* = 0.02, Fig. 5D); however, the relationship between the two measures was not significant for the 3.5 mm circle (*P* = 0.49).

For the HDT experiment, there were no significant changes in RNFL thickness at any eccentricity other than an initial decrease in RNFL thickness at 7 a.m. when going from a seated to a HDT position for the 2.7 mm circle (*P* = 0.036, Fig. 4M). Although changes in RNFL thickness over time were similar between seated and HDT experiments for the 2.7 and 3.5 mm circles (*P* > 0.08), the 4.2 and 4.9 mm circles showed a trend toward greater thinning in the seated condition. These findings suggest that, in addition to BMO-MRW and TRT, a HDT posture diminishes changes RNFL thinning at some eccentricities over the 12-hour period.

### Choroid Thickness

Over the 12-hour period in a seated position, choroid thickness at all four eccentricities gradually decreased, reaching a trough around 11 a.m. to 1 p.m., then rebounded to varying extents (Figs. 3Q–T). For the 2.7 mm and 3.5 mm circles, choroid thickness was significantly reduced relative to baseline at 1 p.m. (*P* = 0.018) and 11 a.m. (*P* = 0.015), respectively. Both the 4.2 and 4.9 mm circles demonstrated significantly reduced choroid thickness at 11 a.m., 1 p.m., 3 p.m., and 5 p.m. (*P* < 0.03). For the HDT experiment, there was no significant change in choroid thickness for any of the four circular scans over the 12-hour period investigated (*P* > 0.55, Figs. 4Q–T).

## Discussion

In most healthy individuals, the neuroretinal rim thins over a 12-hour daytime period, with the majority of change occurring within the first eight hours. This thinning observed in a normal seated position was blunted in a HDT posture.

In a seated posture, the neuroretinal rim was observed to thin 9.55 µm on average over 12 hours, an amount more than double the calculated repeatability of BMO-MRW based on scans obtained within the same session. This gradual thinning was not limited to the neuroretinal rim but also extended into the peripapillary retina, with total retinal thickness measures also thinning out to an eccentricity of about 500 µm from the BMO. We suspect that changes in total retinal thickness are a reflection of changes in the RNFL; this is supported by findings that the RNFL is the dominant component contributing to total retinal thickness at the eccentricities affected (Fig. 1B) and that the circular scans exhibited similar degrees of thinning for RNFL and total retina thickness (∼1 µm).

Although circumpapillary RNFL thickness is generally considered to have excellent repeatability, thinning of the RNFL from morning to evening has previously been reported for healthy individuals.^29^^,^^30^ Overall, the magnitude of this change is small and less than the calculated repeatability for RNFL thickness at the 3.5 mm diameter commonly used for clinical evaluation. Similar to the present study, several previous studies have reported excellent repeatability for BMO-MRW when scans are obtained within short succession.^9^^,^^31^ One study investigated intraday BMO-MRW repeatability for scans obtained at different time points (11 a.m. and 5 p.m.) in patients with glaucoma or ocular hypertension and concluded that the variability in BMO-MRW was minimal.^32^ We found that approximately half the change in BMO-MRW occurred between 7 and 11 a.m., which, in addition to the different cohort of subjects studied, may account for why the authors did not observe greater variability. By quantifying BMO-MRW every two hours over a 12-hour period, we were able to demonstrate that the neuroretinal rim thins throughout the day in a majority of individuals over a period of time when OCT scans would likely be acquired in a clinical setting. Therefore repeatability of BMO-MRW is affected by the time of day at which repeat scans are acquired.^33^ This has important implications for clinical care, because the repeatability of a metric influences its ability to detect pathology. At early stages of disease, the neuroretinal rim changes to a greater extent than peripapillary RNFL thickness.^2^^−^^4^ Although this suggests that BMO-MRW may be better for detecting early disease, there is also greater variability in BMO-MRW than RNFL thickness.^3^ Acquiring measures at the same time of day could potentially maximize repeatability of BMO-MRW, thereby improving our ability to diagnose disease and detect its progression.

The reason for neuroretinal rim thinning over the 12-hour period studied remains unknown, because change in BMO-MRW was not related to changes in IOP, ONSD, MOPP, or other ONH structural changes that could result in rim thinning (e.g., an increase in BMO area or posterior displacement of the ALCS, which could lead to distribution of axons over a larger area or stretching of axons, respectively). However, our finding that rim thinning does not occur in a HDT posture suggests that ONH morphology is influenced by the translaminar pressure difference (i.e., IOP minus CSFP). Although both IOP and ONSD were elevated in HDT, anterior displacement of the BMO suggests that CSFP was likely increased to a greater extent than IOP in this posture, consistent with previous findings.^17^ A potential explanation for the observed BMO-MRW thinning considers habitual changes in body posture over the course of a day. It is possible that the neuroretinal rim increases in thickness during sleeping hours because of the relative elevation in CSFP that occurs in a supine position (i.e., the translaminar pressure difference is lower than in an upright position). There may then be a slow recovery/thinning of the neuroretinal rim after waking up and assuming an upright posture. Although it has previously been demonstrated that there is no change in OCT-derived rim area or volume when transitioning from a supine posture to a seated position, scans were obtained after only a five-minute period of acclimation to each posture.^34^ A recent study in nonhuman primates demonstrated that neuroretinal rim tissue does not change rapidly in response to changes in IOP, but changes over a prolonged period of time.^12^ Therefore changes in ONH morphology may not be immediately evident after a change in pressures, but rather manifest slowly over time. Interestingly, wireless telemetry in nonhuman primates has revealed a slight increase in intracranial pressure around 2 to 3 p.m. while IOP remains fairly stable^15^; if a similar pattern of decreased translaminar pressure difference occurs in humans around 2 to 3 p.m., it could help explain why the BMO-MRW thinning leveled off around the 3 p.m. session. We are unable to determine whether the observed thinning is a reflection of axon diameter, vasculature, glial tissue, or extracellular matrix. However, because intra-axonal swelling of the retinal nerve fibers due to axoplasmic flow stasis is thought to be one of the earliest structural changes in response to elevated CSFP,^35^ it is possible that a similar phenomenon occurs on a subclinical level when individuals are in a supine or HDT posture and recovers in an upright posture. In addition to elevated CFSP, changes in other orbital components may occur in a supine or HDT posture (e.g., orbital tissue, vasculature), although these were not measured. Cerebral venous pressure is known to be elevated in a supine posture relative to a seated position, as evidenced by an increase in internal jugular vein area,^36^ and vascular engorgement or an increase in interstitial fluid may also occur overnight. We propose that the observed changes in BMO-MRW are a direct reflection of the changes in the translaminar pressure difference that occur when an individual transitions from a supine position overnight to an upright posture during waking hours.

Overall, there were considerable interindividual differences for the subjects in this study with regards to the extent of BMO-MRW thinning, with several individuals exhibiting large amounts of thinning (e.g., >20 µm) over 12 hours in a seated position and others showing negligible change. Although an association was found between baseline BMO-MRW and degree of neuroretinal rim thinning, this relationship was only modest in strength (*R*^2^ = 0.34). On average, the neuroretinal rim thinned approximately 8 µm more in females than males over the 12-hour period. Although we cannot determine the precise reason for this discrepancy, it is noteworthy that females are more likely than males to develop idiopathic intracranial hypertension, particularly when obese and of child-bearing age.^37^^−^^39^ The use of oral contraceptives has also been associated with idiopathic intracranial hypertension, suggesting a possible role of female sex hormones in the development of elevated CSFP.^40^^,^^41^ Although it is possible that the female subjects in our study experienced greater increases in CSFP in supine and HDT postures, and therefore greater neuroretinal rim thickening overnight, there was no difference in optic nerve sheath distension in HDT between sexes, suggesting this was not the case. Factors influencing the amount of neuroretinal rim thinning throughout the day remain unknown and are outside the scope of this study; however, we hypothesize that tissue properties of the neuroretinal rim and underlying peripapillary sclera may play a role. The ONH has been described as a biomechanical structure, with material properties of connective tissues being an important factor in ONH response to stresses and strains.^42^ It has recently been demonstrated that compliance of the scleral shell increases in a rat model of menopause, suggesting a possible role of hormones in ONH biomechanics.^43^ Whether hormones influence ONH biomechanics and neuroretinal rim thinning, and could potentially explain differences between sexes in humans, remains unknown. There is likely a range of normal connective tissue phenotypes, which may influence ONH response to translaminar pressures and could be a harbinger for susceptibility to disease.

Remodeling of ocular tissues is known to occur with age and in pathologies such as glaucoma,^44^^,^^45^ and it is possible that pathological eyes may exhibit greater neuroretinal rim variation throughout the day than was observed in our normal cohort. For example, animal studies have shown that the ONH and peripapillary sclera exhibit hypercompliance in early glaucoma, demonstrating exaggerated responses to changes in IOP.^46^ Although tissue remodeling in papilledema is not well established, it is likely that edematous tissue would respond differently to changes in pressures than healthy tissue. In addition to changes in ONH structural properties, patients with glaucoma tend to exhibit greater and more erratic diurnal variations in IOP than healthy controls.^47^ In fact, large diurnal variability in IOP has been suggested to be a risk factor for glaucomatous damage, although this is a topic of debate.^48^^−^^51^ It is possible that assessing diurnal variability of ONH structure in addition to IOP would enhance our understanding of risk of disease. Similar to glaucoma and IOP, patients with idiopathic intracranial hypertension can exhibit wide variations in CSFP over a 24-hour period.^52^ Due to the predicted combination of altered tissue properties and greater fluctuation in pressures, we anticipate that change in neuroretinal rim thickness throughout the day, both under normal conditions and in response to a provocative test such as a HDT or supine body posture, may aid detection of disease. This will be the focus of future investigations.

Before determining whether changes in BMO-MRW have potential applications for disease detection, it will be important to establish repeatability of BMO-MRW change across days. If the observed neuroretinal rim thinning is related to connective tissue properties, we would expect BMO-MRW change to exhibit reasonably good repeatability. However, it is possible that other factors may be involved; for example, if the neuroretinal rim change is a delayed response to alterations in the translaminar pressure difference, the time that an individual wakes up in the morning or the degree of head incline during sleep may influence morphological change. Our lab previously performed a pilot study in which OCT scans and IOP measures were acquired from 7 a.m. to 7 p.m.; eight subjects from the present study also participated in the pilot study (data not shown).^53^ On average, the magnitude of change differed 5.6 µm between the two studies, performed several months apart. Some individuals exhibited either large changes or minimal changes on both days (e.g., −23.2 and −19.1 µm, −2.9 and +3.4 µm, respectively); however, whether these changes represent inherent characteristics of the ONH remains to be determined.

The present study has several limitations. The sample size for the subset of subjects participating in the HDT experiment was fairly small; however, this was unavoidable because of the constraints of the study. Additionally, change in ONH morphology over time was only studied with subjects in two postures: seated and 6° HDT. The study could have been strengthened by including varying degrees of tilt, including a supine position. Additionally, a 24-hour study would add to the findings by providing a more complete picture of diurnal changes in ONH morphology and how they may relate to posture during sleeping hours. Another limitation is that CSFP was not measured directly in our normal subjects due to its invasive nature. While ultrasonography measurement of ONSD allowed us to infer relative increases in CSFP in HDT, there is not a reliable method to translate change in ONSD to change in CSFP; therefore, changes in the translaminar pressure difference could not be quantified. Finally, subjects were allowed seated or standing breaks from HDT totaling up to 36 minutes, which could have reduced the total change ONH morphology. Specifically, the translaminar pressure difference increased (i.e., favored a relative increase in IOP compared to CSFP) during these brief breaks and likely influenced ONH morphology. It has previously been demonstrated that strict adherence to an HDT posture is required for the development of optic disc edema,^54^ and we may have observed more marked differences in ONH structure if subjects had been confined to an HDT posture for the entire 12-hour period.

In conclusion, the ONH neuroretinal rim and peripapillary tissue are not static structures but normally exhibit changes in thickness throughout the day. These normal fluctuations are influenced by body posture, suggesting a role of the translaminar pressure difference. Variations in ONH and peripapillary structures may have important clinical implications, though it remains to be determined whether the degree of change throughout the day is related to risk of disease. Future investigations will study structural variations in pathologies such as glaucoma and papilledema.
